# Transformers for cardiac patient mortality risk prediction from heterogeneous electronic health records

**DOI:** 10.1038/s41598-023-30657-1

**Published:** 2023-03-02

**Authors:** Emmi Antikainen, Joonas Linnosmaa, Adil Umer, Niku Oksala, Markku Eskola, Mark van Gils, Jussi Hernesniemi, Moncef Gabbouj

**Affiliations:** 1 VTT Technical Research Centre of Finland Ltd., 33101 Tampere, Finland; 2 Faculty of Medicine and Health Technology, Tampere University, 33720 Tampere, Finland; 3 Finnish Cardiovascular Research Center Tampere, 33520 Tampere, Finland; 4 Vascular Centre, Tampere University Hospital, 33520 Tampere, Finland; 5 Tays Heart Hospital, Tampere University Hospital, 33521 Tampere, Finland; 6 Faculty of Information Technology and Communication Sciences, Tampere University, 33720 Tampere, Finland

**Keywords:** Cardiovascular diseases, Information technology, Machine learning

## Abstract

With over 17 million annual deaths, cardiovascular diseases (CVDs) dominate the cause of death statistics. CVDs can deteriorate the quality of life drastically and even cause sudden death, all the while inducing massive healthcare costs. This work studied state-of-the-art deep learning techniques to predict increased risk of death in CVD patients, building on the electronic health records (EHR) of over 23,000 cardiac patients. Taking into account the usefulness of the prediction for chronic disease patients, a prediction period of six months was selected. Two major transformer models that rely on learning bidirectional dependencies in sequential data, BERT and XLNet, were trained and compared. To our knowledge, the presented work is the first to apply XLNet on EHR data to predict mortality. The patient histories were formulated as time series consisting of varying types of clinical events, thus enabling the model to learn increasingly complex temporal dependencies. BERT and XLNet achieved an average area under the receiver operating characteristic curve (AUC) of 75.5% and 76.0%, respectively. XLNet surpassed BERT in recall by 9.8%, suggesting that it captures more positive cases than BERT, which is the main focus of recent research on EHRs and transformers.

## Introduction

Electronic health records (EHRs) encompass evidence of patient care paths and outcomes. Different EHR models have been widely adopted by healthcare facilities and continue to accumulate increasing amounts of data with potential to discover new medical knowledge and to support decision making to improve outcomes for new patients^1^. Although EHRs offer large volumes of longitudinal real-life data for improved machine learning (ML), they still challenge the methodology with their heterogeneous, sparse, often incomplete and even erroneous data^2^. Moreover, due to the sensitive nature of the data, privacy issues and regulations will further complicate model development and deployment in the future^3^. Some regulations may require database anonymization to protect data privacy but this may result in decreased data quality due to additional noise and gaps.

Cardiovascular diseases (CVDs) have held their ranking as the leading cause of death worldwide for years and continue to impose an increasing challenge to the global health. In 2017, CVDs alone caused 17.8 million deaths globally, showing an alarming 21.2 % increase in the yearly CVD death count since 2007^4^. Furthermore, CVDs can be a risk factor in relation to other diseases and increase the demand for hospital care. For instance, they have been linked with poor prognosis in the context of COVID-19, which threatened health care capacity all over the world^5^. The problem of CVDs has not been sufficiently addressed. While the risk could be efficiently reduced with lifestyle changes towards physically active lives and healthier diets, the reports of the aging population and overwhelming obesity rates indicate enduring prosperity for CVDs^4^. Predictive models may help identify high-risk patients and patient deterioration and may be used to focus healthcare resources efficiently to improve patient outcomes and manage the increasing CVD counts. Data-driven approaches are expected to renew the clinical cardiology practice, ascertain their place in the clinician’s toolbox, and to reform our understanding of the causes of CVDs^6,7^.

Transformer neural networks are the state-of-the-art machine learning methods for sequential data modelling. Developed for natural language processing, their built-in properties respond to many needs that arise when using EHR data. Thanks to them combining attention and positional encoding, transformers can be applied to learn bidirectional temporal dependencies despite the sparsity and possible errors in the large volumes of EHR data. Their design to handle textual input does not exclude numerical input and may thus be useful for heterogeneous input types. In the context of EHRs, they have been applied mainly to clinical notes or diagnoses^8–11^. Yet, their capabilities to capture more complex dependencies in heterogeneous databases have received little attention^12^. Furthermore, prior studies have focused on one transformer variant; bidirectional encoder representations from transformers (BERT)^13^. A newer model, XLNet, has surpassed BERT in many baseline natural language processing tasks^14^. This work uses an anonymous cardiac patient EHR database to compare the learning capabilities of BERT and XLNet in the important application of mortality risk prediction. Here, the transformer models are applied to multi-modal heterogeneous patient event time series, comprising both textual and numerical attributes.

Prior to transformers, convolutional neural networks (CNNs) and recurrent neural networks (RNNs) achieved encouraging results in, e.g., arrhythmia detection from electrocardiograms (ECG), diagnostic decision support using cardiovascular images, and diagnosis prediction from EHR data^15–18^. The introduction of attention mechanisms provoked countless new studies reporting improved results^19–23^. Importantly for clinical applications, attention gave interpretability to the model outcomes, thus offering one solution to the primarily criticized shortcoming of deep learning (DL) methods^19^. For example, Choi et al. presented the RETAIN model which coupled attention with recurrent neural networks (RNNs) to predict heart failure from EHR data. They presented a method for prediction interpretation while reporting an 87% area under the receiver operating characteristics curve (AUC)^21^. Another relevant study was conducted by Rajkomar et al. who used an ensemble of three DL models, one of which was attention-based, and tested their system on EHR data from two hospitals. They achieved 93–95% AUC for in-patient mortality prediction at 24 h after admission^22^.

The original Transformer relied exclusively on attention mechanisms^24^. The Transformer and its variants surpassed RNNs by allowing parallelized computing and by learning bidirectional dependencies. They learned longer-range dependencies at improved training time, which is crucial with long input sequences like EHR histories^24^. The first studies applying transformers directly on EHRs were built on BERT, which bases its learning strategy on masking the input^13^. Shang et al. combined BERT with ontology embeddings from a graph neural network creating G-BERT for medication recommendation^10^. They reported a 1% increase in precision-recall AUC as compared to RETAIN. BEHRT by Li et al. applied BERT directly for disease prediction by using sequences of diagnoses available in the EHRs^9^. They reported a patient-averaged AUC of 95–96% for varying prediction windows extending up to 12 months. Thirdly, Rasmy et al. reported up to 2% improvement in disease prediction with their Med-BERT as compared to RETAIN^11^. They evaluated Med-BERT for heart failure prediction in diabetic patients and pancreatic cancer onset prediction. Some studies have additionally proposed somewhat modified transformers for EHR representation learning^25–27^.

In this study, we apply the ground-breaking transformer models on patient time series to predict 6-month mortality in cardiac patients. The 6 months prediction period may offer actionable predictions for many chronic conditions. Unlike BEHRT and Med-BERT which were trained on sequences of diagnostic codes, we incorporate over a dozen different event types each described by multiple attributes to capture a more complete depiction of the patient history. By feeding the transformers sequences of patient events with timestamps based on age, the models may learn how the interplay between different events and their outcomes, as well as temporal dependencies, affect the patient outcome. With this approach, the patterns learned by the model may unveil unforeseen associations between different events. Moreover, we study both BERT and XLNet. Unlike BERT, XLNet is an auto-regressive transformer variant that avoids corrupting the input^14^. We exploit the anonymous EHRs of over 23,000 cardiac patients who were treated at Tays Heart Hospital in Finland and report our findings on using privacy-preserving anonymous data in model development, an increasingly common starting point for future EHR studies. A previous machine learning study on the same database considered a subset of 9066 consecutive acute coronary syndrome patients and achieved an AUC of 89% for 6-month mortality using conventional, non-deep learning methods^28^. This study takes up a more complex challenge of predicting mortality in all available CVD patients, comprising a more heterogeneous patient population.

## Methods

### Study data

The longitudinal study data comprised three Finnish data sources: (1) the EHR by the Pirkanmaa Hospital District (PHD), (2) the KARDIO registry by the Tampere Heart Hospital, and (3) the Finnish mortality registry by Statistics Finland. The PHD EHR extend back to the 1990’s and the date of death from the mortality registry was included for the matching period. The PHD EHR data include hospital discharge diagnoses, which record every diagnosis recorded for the patient in ICD-10 format (and previously in ICD-9 and ICD-8 formats). This data is equally reported in every hospital nationally and the validity of the registry is high for many significant cardiovascular conditions such as strokes, coronary heart disease and heart failure^29–31^. The KARDIO registry is the most recent of the three; its first entries date back to the early 2000’s. The original database was automatically collected from the three registries until January 2020 as a part of a retrospective registry study, MADDEC (Mass Data in Detection and Prevention of Serious Adverse Events in Cardiovascular Disease)^32^.

The study was approved by the Pirkanmaa Hospital District Institutional Review Board’s scientific steering committee. Informed consent is waived since the retrospective nature of the study by the Pirkanmaa Hospital District Institutional Review Board’s scientific steering committee. The study was conducted according to the declaration of Helsinki as applicable and the study data was processed in accordance with the Finnish legislation. An anonymous version of the database was used, comprising 72,680 patients (9172 deceased patients within six months of their last visit, i.e., 12.6%) all treated at the Tays Heart Hospital for different cardiac conditions.

### Input sequence formulation

**Figure 1:**
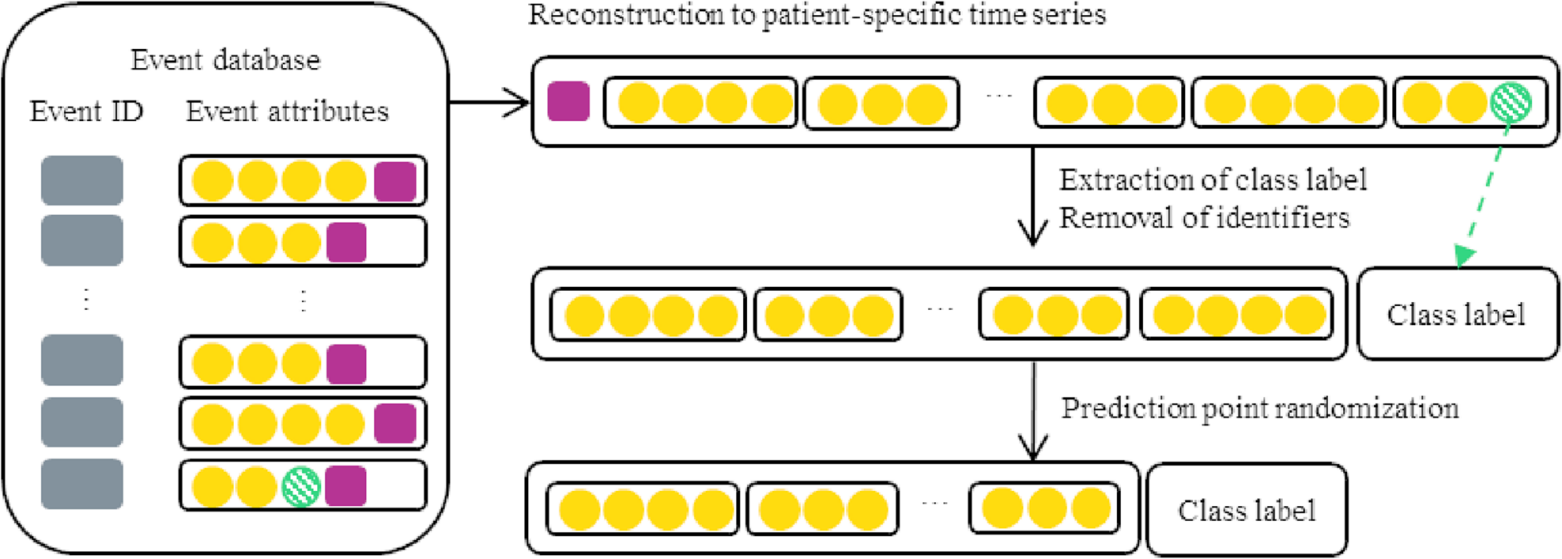
A schematic example of how a patient’s events were formulated into a time series. The records in the event-oriented database contain the event type specific attributes (yellow circles). First, the events related to the same patient ID (magenta square) were combined to a sequence sorted according to their temporal attributes. Hereafter, the patient ID was no longer necessary. Next, the class (prediction target, i.e., death within six months or alive) was computed using the death-related attribute (green striped circle) and the time between that and the previous event. Finally, the point of prediction was randomized. If the class was positive, the time to death from the final remaining event was maintained within the selected six month period.

The patient records were extracted from the event-oriented database, pre-processed, and finally ordered on temporal attributes to formulate a single time series of events for each patient. The resulting time series were further processed into appropriate input for the transformer neural networks, as summarized in Fig. 1. In the anonymous database, the temporal attributes were age-based (on a daily level, i.e., days since birth) and the real dates and times were unknown.

Data pre-processing included replacing Roman numerals with Arabic numerals, filling in event start or end times when only one of the two was missing, and unifying notations. Units and measurement names, anesthesia types, and urgency classes were translated from Finnish to English. Additionally, some body-mass index (BMI) values below 0.02 were presumed to use centimeters instead of meters for height, and thus, multiplied by $10^4$ to restore correct units. For events where the ending time preceded the recorded event start time, the timestamp order was presumed a typographical error and the timestamps were switched. In the end, only the pre-processed event start time was included as the event durations were generally error-prone.

Any events occurring before the patient became of age were excluded. Additionally, any events with missing event timing or overlap with the date of death were excluded. The latter consists of events extending to the date of death, e.g., resuscitation or procedures, or beyond, such as lab values or diagnoses.

**Table 1:** Event specific attributes

Event type	Attributes
Labs	Event type, start time, residence, lab test value (num), lab test value (char), lab test name, lab test unit
Diagnosis	Event type, start time, residence, diagnosis code$^{\rm a}$, diagnosis priority
Medication	Event type, start time, residence, ATC code$^{\rm b}$, daily dosage, dose unit, administration method
Operation	Event type, start time, residence, sequence number, code ID, code$^{\rm c}$
Procedure	Event type, start time, residence, anesthesia type, ASA class$^{\rm d}$, operation urgency
Measurement	Event type, start time, residence, measurement value (num), measurement context*, measurement name, measurement unit, measurement context code**
Hospital visit	Event type, start time, residence
Hospital ward	Event type, start time, residence, times repeated, ward
Angiography	Event type, start time, residence, times repeated, ward, primary angiography findings, sex, stenosis (boolean), primary puncture places
Percutaneous coronary intervention (PCI)	Event type, start time, residence, times repeated, ward, complications, sex, indication, urgency
Imaging	Event type, start time, residence, imaging type
Coronary care unit (CCU)	Event type, start time, residence, times repeated, ward, dialysis, sex, temporary pacemaker, primary vasoactive medication
Transcatheter aortic valve implantation (TAVI)	Event type, start time, times repeated, ward, dyslipidemia, fluoroscopy time, sex, glomerular filtration rate, hypertension
Resuscitation	Event type, start time, residence, times repeated, ward, family history***, sex, hypertension, smoking

*Measurement context in text format, for example a suspected diagnosis or type of the visit.

**Measurement context related code (e.g. an ICD-10 diagnostic code).

***Family history (for early coronary artery disease) was positive if at least one of the patient’s first degree relatives had suffered a myocardial infarction or underwent coronary revascularization (PCI or coronary artery bypass surgery) at an early age ($<55$ and $<65$ years in men and women, respectively).

^a^International Statistical Classification of Diseases and Related Health Problems, the 10th revision (ICD-10).

^b^Anatomical Therapeutic Chemical (ATC) code.

^c^Nordic Classification of Surgical Procedures (NCSP).

^d^American Society of Anesthesiologists (ASA) classification of physical status.

The data sources contained 14 distinct types of events each described by a different set of attributes, as presented in Table 1. Here, we took the liberty of excluding any attributes that were never present for an event type. For angiography, percutaneous coronary intervention (PCI), coronary care unit (CCU), transcatheter valve implantation (TAVI), and resuscitation events, the attributes were limited to the nine most available attributes (out of tens of attributes) to control input sequence length and to fit multiple events in the input sequence. Each event was constructed into a sequence simply by listing the event type and the corresponding attributes in one sequence. Thus, each event type was represented by a specific “sentence structure” mimicking natural language. Any missing attribute values were filled in with ’None’. All event representations started with the event type name, the event starting time, and residence (among Finnish counties) when available. Residence was available in 67%, 74%, and 79% of CCU, resuscitation, and hospital ward events respectively, while it was missing completely for TAVI and in 59–94% for other event types. Event type, start time, all operation attributes, times repeated, ward, sex, stenosis, imaging type, dialysis, temporary pacemaker, primary vasoactive medication, fluoroscopy time, and glomerular filtration rate attributes were all fully available for the relevant event types. The remaining attributes in labs were available in 72–75% of lab events (except textual values only in 1%). Diagnosis code and priority were available in 36% and 41% of diagnosis events respectively, and anesthesia type, ASA class, and urgency in 56%, 61%, and 93% of procedures. All measurement event attributes were available in 91–100% of measurement events. The remaining attributes in angiography, PCI, CCU, and TAVI were available in 98-100% of the respective events, whereas the other attributes for resuscitation events were available in 89% of resuscitation events.

**Table 2:** Pre-processed study data.

	N	Female	Male	Age range	Mean years of data (SD)	Mean no. of events (SD)
Positive	3771	691 (18.3%)	1183 (31.4%)	18–102	6.5 (3.4)	1755 (2364)
Negative	53,606	7249 (13.5%)	11,365 (21.2%)	18–105	4.2 (3.8)	553 (1091)
Total	57,377	7940 (13.8%)	12,548 (21.9%)	18–105	4.4 (3.9)	632 (1255)

The percentages depict the proportion of the (known) sex with respect to the full number of patients on the same row.

*SD* standard deviation.

The individual pre-processed events were combined in order of occurrence into one sequence per patient, forming the patient event timeline. Until this point, the events were linked via patient and event pseudo-identifiers. The pseudo-identifiers were removed and the date of death was isolated and transformed into a binary class: positive (1) when the date of death occurred within 182 days of the last event, and negative (0) otherwise. The date of death was comprehensively obtained from the Finnish mortality registry. Importantly, in the real-life clinical use-case the model could be used to produce predictions at any time of a patient timeline. Therefore, to produce realistic evaluation of model performance and avoid bias due to the retrospective nature of the data, a random number of events at the end of a patient’s timeline were erased. The number of erased events was selected randomly between zero and a patient-specific maximum number such that at least five events remained for the patient, and the death for any positive case would still occur within the selected cutoff from the final remaining event.

Finally, the input sequences were tokenized and the special tokens for class (CLS) and sentence separation (SEP) were added once to each patient timeline according to their expected position in BERT and XLNet^33^. Any numerical input was transformed into string-type integers for tokenization. The age in days was transformed into full years.

### Hyperparameter optimization

The model hyperparameters were optimized using Population Based Training (PBT)^34,35^. PBT is an evolutionary algorithm, which trains several networks with varying hyperparameters in parallel. During the training process, each network can explore hyperparameters randomly in a predefined space or exploit another better performing parallel model by copying its parameters and continuing to explore new hyperparameters with the partially trained model, without restarting the training from scratch.

PBT was applied to optimize the learning rate, dropout fraction, and model dimensions including the number of heads and layers, as well as layer size. Due to memory limitations, only batch sizes 16 and 24 were tested. PBT was run for both BERT and XLNet for 30 epochs on 12 trials with the perturbation interval of ten epochs. Similarly to the original transformers, Gaussian Error Linear Unit (GELU) was used for activation.

### Model evaluation

Eighty percent of the study data was used for model development, while 20% was held out as a test set. The development data was further split into training and validation data, comprising 80% and 20% of the development set, respectively. Stratified splits were used to maintain a similar distribution of positive and negative cases in each set. The data sets were further balanced by taking a random sample of negative cases to match the number of positive cases (see details in Implementation). Model performance was assessed with AUC, precision (positive predictive value), and recall (sensitivity)^36^.

PBT was performed on the development set. The top-performing BERT and XLNet models were validated using stratified fivefold cross validation with the development data. Subsequently, the final BERT and XLNet models were trained with the selected hyperparameters on the full development data and evaluated on the held out test data set.

The final model training was repeated five times to account for the effect of random initialization. Early stopping was applied when the training loss failed to improve at least by 0.0045 over 5 epochs (min_delta and patience in keras EarlyStopping, selected based on the previously observed cross-validation losses). To interpret what the final models had learned, the models were fed example time series from the test set and the attention weights were visualized using BertViz^37^. Min–max normalization was applied to the attention layers prior to the visualization to properly highlight where attention was at its highest and lowest.

### Implementation

The data were tokenized using pretrained tokenizers (bert-base-cased, xlnet-base-cased) available in the Hugging Face model database^13,14^. The transformer models were implemented in Python by using the Hugging Face Transformers library together with Tensorflow^33,38^. The Ray Tune package (function API) was used for hyperparameter optimization^35^. The data split for model evaluation was obtained using scikit-learn^39^. The final models were trained using an Adam optimizer with an epsilon of $10^{-8}$. The sequence length was restricted to 512 tokens such that the latest information in the patient history was included. Overlength sequences were truncated and under-length sequences padded using the tokenizer-specific padding token.

Class imbalance was managed by (1) down-sampling the negative examples in the training and validation sets and (2) using a weighted binary cross-entropy loss function. To ensure that each limited-size batch had a reasonable chance of including some positive cases, the negative samples were randomly down-sampled so that 25% of the samples in both training and validation set were positive. By limiting the extent of down-sampling, the related data loss was also limited. The remaining imbalance was counteracted via the loss function using balanced class weights; each class was weighted by its inverse prevalence in the development set, further divided by the number of classes (two).

A 32 gigabyte Tesla V100-DGXS graphics processing unit (GPU) was used in hyperparameter optimization and training the models.

## Results

**Figure 2:**
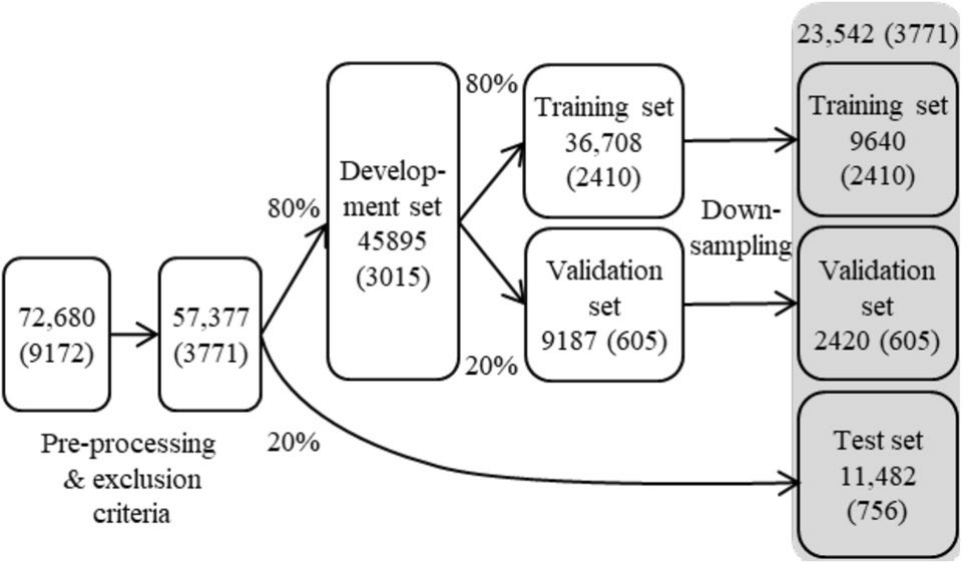
Patient flow diagram. The total number of patients is indicated for each step and the number of positive cases is denoted in brackets. The final data used in model evaluation comprised 23,542 patients and is depicted on a gray background.

Implementing the exclusion criteria reduced the study data from 72,680 patients to 57,377 adult patients, including 3771 (6.57%) positive cases. The demographic details are described in Table 2. The average age of patients was 65 years (79 for positive cases). The sex of the patient was only available for 35.7%, most of which (61.2%) were male. The notably large portion of sex information was lost upon anonymization as the national identification numbers were removed.

After down-sampling the development sets to counteract class imbalance (as detailed in “Methods”), the resulting training and validation sets contained 9640 and 2420 patients (12,060 in total with 3015 positive cases). The test set comprised 11,482 patients with the number of positive cases, 756 (6.58%) corresponding approximately to the prevalence in the full pre-processed data. Thus, the study involved 23,542 individuals (including all 3771 positive cases). The patient flow is summarized in Fig. 2.

The hyperparameters optimized using PBT are presented in Table 3. BERT performed best on learning rates around $5\times 10^{-7}$ to 1$\times 10^{-6}$, whereas rates an order of magnitude larger (${5\times 10^{-6}}$ to ${1\times 10^{-5}}$) worked best for XLNet. The selected configurations comprised 108,312,578 trainable parameters for BERT and 5,482,130 parameters for XLNet.

**Table 3:** Hyperparameters optimized via population based training.

Hyperparameter	BERT	XLNet
Hidden size	144	144
Number of layers	12	6
Number of attention heads	12	6
Feed-forward layer hidden size	128	128
Learning rate	$1\times 10^{-6}$	$5\times 10^{-6}$
Batch size	16	16
Dropout	0.5	0.4

The models with optimized hyperparameters were cross-validated using 5-fold validation to assess their sensitivity to the selection of training instances. The validation results are presented in Table 4. The models achieved similar average AUC. BERT achieved slightly higher precision but the variance between folds was also higher. However, less than half of the predicted cases were true positive cases. Finally, XLNet reached a notably higher average recall, with low variance between folds. Thus, the optimized XLNet was more sensitive to detect positive cases than BERT.

**Table 4:** 5-fold cross-validation of optimized models.

Fold	BERT			XLNet		
	AUC	Precision	Recall	AUC	Precision	Recall
1	0.7452	0.4567	0.8126	0.7438	0.4592	0.8027
2	0.7366	0.5244	0.6783	0.7570	0.4740	0.8159
3	0.7703	0.4711	0.8640	0.7692	0.4873	0.8292
4	0.7432	0.5350	0.6849	0.7454	0.4496	0.8292
5	0.7689	0.5047	0.7993	0.7557	0.4815	0.7977
Mean	0.7528	**0.4984**	0.7678	**0.7542**	0.4703	**0.8149**

Performance metrics in the validation set, after 50 epochs.

The best mean score for each metric (AUC, precision, recall) is in bold.

The final model training was repeated five times to examine the effect of random initialization. The test results obtained on the held-out test set are presented in Table 5. The corresponding mean specificity scores were 78% and 69% for BERT and XLNet, respectively. The test set results support the observations from cross-validation. The slight improvement in AUC and recall were likely due to early stopping, which stopped the training already before 50 epochs in all cases. This prevented over-fitting, which occurred remarkably early for this data and models. The drop in precision is explained by the increased class imbalance in the test set but also underlines that both models produced mostly false positives, despite capturing 73–83% of the positive cases on average. In comparison to BERT, the improvement in XLNet’s recall exceeds the drop in precision and, thus, the XLNet model may be more useful.

**Table 5:** Blind test results on five different initializations.

Run	BERT			XLNet		
	AUC	Precision	Recall	AUC	Precision	Recall
1	0.7398	0.2248	0.6336	0.7556	0.1533	0.8373
2	0.7612	0.1923	0.7421	0.7602	0.1574	0.8360
3	0.7586	0.1919	0.7355	0.7654	0.1665	0.8201
4	0.7547	0.1937	0.7209	0.7609	0.1601	0.8280
5	0.7591	0.1571	0.8333	0.7586	0.1563	0.8347
Mean	0.7546	**0.1919**	0.7330	**0.7602**	0.1587	**0.8312**

The best mean score for each metric (AUC, precision, recall) is in bold.

**Figure 3:**
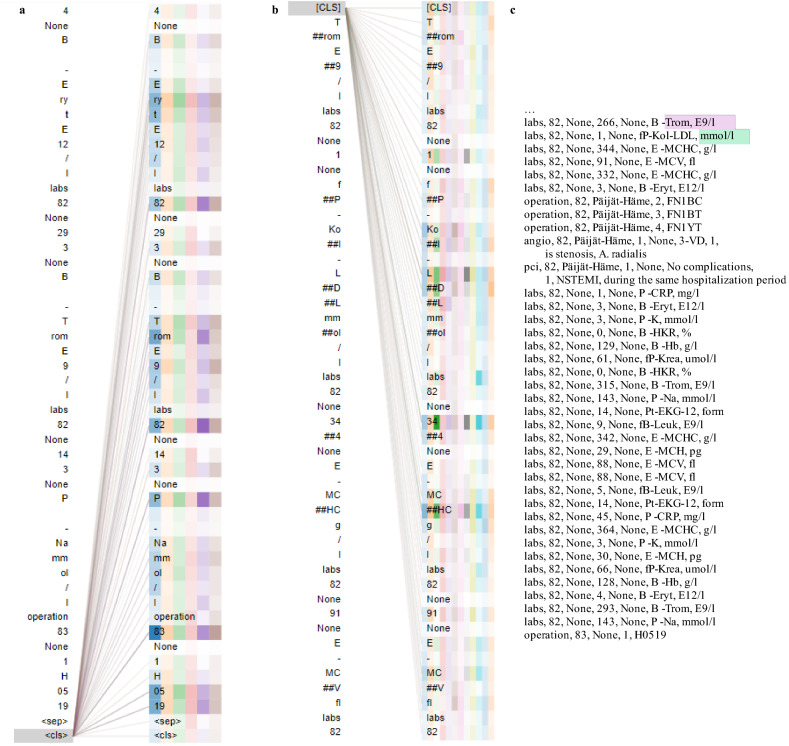
Attention (**a**) in the fifth attention layer in XLNet at the end of an example subsequence near the <cls> token, and (**b**) in the final attention layer in BERT at the start of an example subsequence near the [CLS] token. The different colours represent the (**a**) six and (**b**) 12 attention heads; the more opaque the colour, the heavier the attention. The final events of the example time series are presented in a human readable format in (**c**), where the first information visible to BERT is highlighted with purple and with green for XLNet. Figures (**a,b**) were produced using BertViz^37^.

The final BERT and XLNet models exhibited very similar metrics (run number 5 in Table 5) and were fed an example time series from the test set for interpretation. The model attention for the 50 tokens nearest to the classification token in an example time series are depicted in Fig. 3a,b for XLNet and BERT, respectively. The selected (full sequence) example was correctly labeled positive by XLNet and mislabeled negative by BERT. The corresponding pre-processed example input before tokenization is depicted in Fig. 3c. The full patient history comprised 111 events, whereas the models could only consume input from up to 38 events.

The 83 years old patient’s latest event was an operation encoded as H0519, which stands for a simulation film, possibly related to radiation therapy planning. Their history also showed, e.g., an angiography of the heart and/or coronary artery, a percutaneous transluminal coronary angioplasty, and an intraventricular stent placement to enlarge the coronary artery, all within the past year. As seen in Fig. 3a at the end of the sequence, XLNet attends especially to the age (three instances visualized) and to the operation code. Most other layers also attend to age and the operation code at the <cls> classification token, while exhibiting varying attention to the other inputs. In contrast, BERT’s attention at the [CLS] classification token in Fig. 3b does not exhibit special attention to the patient’s age (not the primary focus of attention in any layer) but attends to some of the lab results. It is noted that a tokenizer specialized in EHR data might not only make the interpretation easier but also improve attention results.

## Discussion

This work explored and compared the potential of two popular transformers, BERT and XLNet, in the task of predicting 6-month mortality in cardiac patients at randomly chosen events recorded in their EHR. The heterogeneous electronic health record data were constructed into semi-structured multi-event time series to exploit the temporal information. We achieved a higher recall with XLNet, suggesting that it captures more positive cases than BERT. It has been argued that the learning strategy implemented in XLNet is better capable of capturing long-term dependencies in sequences^14^. To our knowledge, this is the first study exploring XLNet for mortality prediction from electronic health records.

Previous studies often set their focus on in-patient mortality within 24 h of admission, which can be especially beneficial for applications at intensive care units^2^. In contrast, patients with long-term conditions may profit from earlier predictions. The 6-month prediction period selected in this study allows time for clinicians to re-evaluate the patient’s needs and make their care more effective to decrease their risk of death. It provides time for any additional tests and diagnostics, as well as a realistic possibility for interventions to take effect. Six months was considered a suitable period to explore model performance in such a heterogeneous cardiac patient population.

As compared to a prior study using extreme gradient boosting (XGBoost) on the same database and prediction target, the presented results fall short of the previous AUC result^28^. This may, however, be expected because the prior study focused on a specific homogeneous patient group (with acute coronary syndrome) whereas the current work with a larger portion of the database included a wide heterogeneous spectrum of CVD patients. Moreover, the more refined and smaller subset of data in the previous study allowed for features selected by expert clinicians, which may have further facilitated good performance but also increased manual work. Additionally, this study used the anonymous database, which lead to more noise and gaps in the training data and only offered dates relative to a patient’s birth instead of real dates. Hence, the importance of the concurrent planning of the analysis and anonymization is underscored. In this study, because the collection of study data was terminated on a specified date without any follow-up, the data contained patients that were still in care or did not have a full six months since their last event. These examples could not be filtered from the anonymous data as the real dates were no longer available and, thus, they may cause the model to be too optimistic about patient survival. The missing real dates also prevented the analysis of time-dependent differences between patient timelines which might exist due to, e.g., updates in care guidelines. Moreover, the sex of patient was largely missing although it is an important clinical factor affecting patient outcomes.

Even though some transformers such as XLNet are in principle able to consume sequences of any length, the models are still limited by the memory resources of the hardware used for training and visual output interpretation. This poses a challenge for incorporating all different event types and their attributes from the patient history. Here, the 512 tokens representing the most recent events of the patient were used while the captured time period varied. Formulating the EHR data as multi-event time series may facilitate the extraction of new knowledge concerning the role and relationships of different types of events. Future research may explore longer input sequences with XLNet or alternative ways to incorporate multi-event information. For instance, replacing code based attributes with full text descriptions may improve performance but would require longer input sequences to feed the model the equivalent portion of patient history. In the future, harmonization of hospital information management systems may additionally yield better grounds regarding the selection of attributes as they are inherited from the hospital’s original system. Further improvement may be achieved by using tokenizers specially trained on clinical data or pre-trained transformers. Here, due to the lack of such resources for XLNet, both models were trained from scratch to facilitate a fair comparison. Notably, our results show that the standard English tokenizers can produce promising learning results.

As demonstrated in this work, transformers provide a means to interpret individual outputs and the predictions may therefore become a valuable part of the clinical workflow and answer to the requirements set for ML models in CVD predictions^40^. Nevertheless, intuitive and user-friendly output interpretation interfaces for clinicians need further development so that this capability can be properly harnessed. The resulting tools may be efficiently integrated to the EHR system itself, although additional computing resources are likely required.

## Conclusion

Using transformers to learn bi-directional dependencies in EHRs shows promise in mortality prediction, despite the sparsity of the data. We compared BERT and XLNet for CVD patient mortality risk prediction from EHR data. While prior research has focused on BERT for EHR applications, the results of this study suggest that future studies may achieve improved results using XLNet. Similar models with actionable outputs, as presented here, could improve patient outcomes with chronic diseases, such as CVDs, and be directly integrated to the EHR systems for everyday clinical use.

We also observed that transformers may perform better in more refined patient groups. The wide spectrum of CVD patients in this study added complexity to the prediction problem, producing weaker performance as compared to conventional machine learning in a more refined patient group. Furthermore, more concise representations have reached better learning results, whereas the multi-attribute multi-event representation faces computational restrictions. Hence, in the future, improved results may be obtained via more sophisticated representations, transfer learning from pre-trained models, or via improved computational power. As in the presented study, anonymous data will become an increasingly common basis for model development. In such cases, the performance of data-driven models may benefit from an improved anonymization process.

## Data Availability

The anonymized data is available for scientific purpose upon reasonable request to J.H. (jussi.hernesniemi@sydansairaala.fi) pending the approval of the MADDEC study steering committee.
